# A One-Year Randomized Controlled Clinical Trial of Three Types of Narrow-Diameter Implants for Fixed Partial Implant-Supported Prosthesis in the Mandibular Incisor Area

**DOI:** 10.3390/bioengineering11030272

**Published:** 2024-03-12

**Authors:** Ji-Ho Ahn, Young-Jun Lim, Jungwon Lee, Yeon-Wha Baek, Myung-Joo Kim, Ho-Beom Kwon

**Affiliations:** 1 Department of Prosthodontics and Dental Research Institute, School of Dentistry, Seoul National University, Seoul 03080, Republic of Korea; jihoahn31@gmail.com (J.-H.A.); silk1@snu.ac.kr (M.-J.K.); proskwon@snu.ac.kr (H.-B.K.); 2 Department of Periodontology, One-Stop Specialty Center, Seoul National University Dental Hospital, Seoul 03080, Republic of Korea; jungwonlee.snudh@gmail.com; 3 Department of Prosthodontics, Gwanak Center, Seoul National University Dental Hospital, Seoul 08826, Republic of Korea; obero7@snu.ac.kr

**Keywords:** narrow-diameter implants, anterior mandible, immediate loading, primary stability, marginal bone loss

## Abstract

Narrow-diameter implants (NDI) serve as a solution for treating limited bone volume in the anterior mandible. This study aimed to evaluate the one-year clinical outcomes of various NDIs in the mandibular incisor area after immediate loading in partially edentulous patients. This single-center, prospective, single-blinded, randomized controlled trial study included 21 patients, with 7 patients in each of the following groups: control (BLT NC SLActive^®^; Straumann), experimental group 1 (CMI IS-III Active^®^ S-Narrow; Neobiotech), and experimental group 2 (CMI IS-III Active^®^ Narrow; Neobiotech). Using full digital flow, two fixtures were placed in each patient and immediately provisionalized on the day of surgery. Evaluations encompassed periapical radiographs, implant stability quotient (ISQ), implant stability test (IST) readings, per-implant soft tissue health, patient satisfaction surveys, and esthetic score assessments. Definitive prostheses were delivered twelve weeks post-surgery (CRiS, number: KCT0007300). Following exclusions due to low stability values (*n* = 2), fixture failure (*n* = 5), and voluntary withdrawal (*n* = 1), the implant success rate for patients completing all clinical protocols stood at 100%. The resulting patient failure rates in the control, experimental group 1, and experimental group 2 were 50.0%, 42.9%, and 14.3%, respectively. There were no significant differences between the groups in terms of marginal bone loss, soft tissue health, patient satisfaction, and esthetic scores. Narrow implants showed superior clinical outcomes, followed by S-Narrow and Straumann implants. Calculated one-year survival rates at the implant level were 66.7% for the control group, 85.7% for experimental group 1, and 100% for experimental group 2. All three types of NDIs showed acceptable clinical and radiographic results during the year-long observation period.

## 1. Introduction

Replacing mandibular incisors with implants presents challenges due to their small dimensions. Mandibular incisors have the smallest root size and surface area [1]. Following extraction, bone reduction occurs, especially in the buccolingual direction, due to remodeling. The anterior region of the mandible is more vulnerable to bone resorption because it consists of trabecular bones and thin buccal bones [2]. Augmentation techniques for bone and soft tissue compensation often result in implant failures [3].

Narrow-diameter implants (NDIs) offer a solution for limited bone volume without additional augmentation. The general consensus is that implants with a diameter ≤ 3.5 mm are commonly referred as NDIs, which are further divided into three categories (Category 1, <3.0 mm; Category 2, 3.0–3.25 mm; Category 3, 3.30–3.50 mm) [4]. Indications for NDIs include cases with reduced interradicular bone, narrowed buccolingual ridge width, or limited mesiodistal prosthetic space [5,6]. Compared to standard-diameter implants, NDIs alleviate the costs, time, and pain associated with bone and tissue augmentation procedures [7]. NDIs also secure the required distance between the implants and natural teeth. The literature has shown that the long-term survival and success rates of NDIs match those of standard diameter implants [6,8,9,10,11,12].

Advances in digital technology enable the combination of cone-beam computed tomography (CBCT) and intraoral scanning. Full digital flow allows a “top-down” approach where implant position is determined by the virtual definitive prosthesis position. Using computer-aided design (CAD) software, both esthetic and stability aspects are considered during implant position planning by assessing definitive prosthesis contour and residual bone dimensions. Surgical templates are then created through computer-aided design and manufacturing (CAD/CAM) processes, which now have become accessible and affordable.

The careful planning of implants with adequate initial stability permits immediate restoration, comparable to conventional loading results [13,14,15]. Immediate loading is particularly favored by patients missing anterior teeth due to social and esthetic considerations. Studies indicate that soft tissue contours around immediately placed and restored prostheses yield satisfactory esthetic outcomes [16,17].

The CMI IS-III Active^®^ S-narrow fixture (NeoBiotech Co., Seoul, Republic of Korea) was specifically developed for the anterior region of the mandible. With its “super narrow” diameter (3.2 mm), it simplifies implant placement in the narrow ridge of the anterior mandible without additional procedures. Few randomized controlled trials have compared the clinical outcomes of NDIs in the anterior mandible based on different dimensions.

This study investigated the implant survival rate, success rate, and peri-implant marginal bone loss of three different types of NDIs in the mandibular incisor area of partially edentulous patients undergoing immediate loading. Secondary outcomes included peri-implant soft tissue health, patient satisfaction, and esthetic scores.

## 2. Materials and Methods

### 2.1. Clinical Study Design

**Figure 1:**
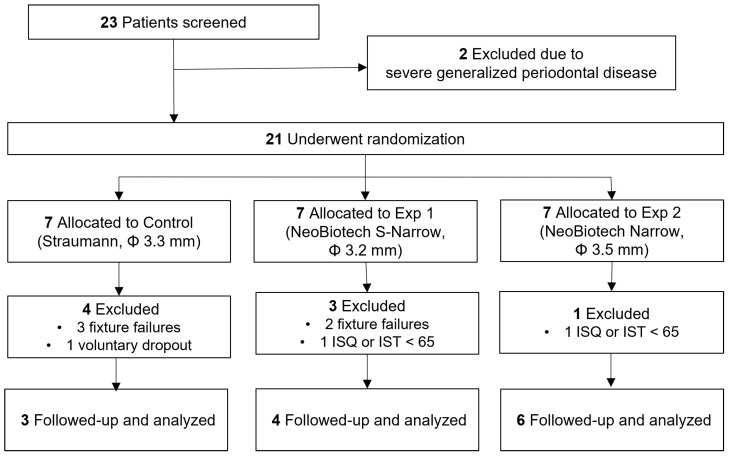
Flowchart of the study protocol.

This study was a single-center, prospective, single-blinded, randomized controlled trial. The study enrolled individuals who required the extraction of two or more mandibular incisors, with no systemic diseases. These patients were randomly assigned to one of three groups (Figure 1) and were kept blinded to the type of implants they received. The patients were recalled for clinical and radiographic evaluations after surgery. The inherent characteristics of the intervention made it impossible to conceal product information from healthcare professionals. The BLT NC SLActive^®^ (Straumann, Basel, Switzerland) implants were 3.3 mm in diameter and 10.0 or 12.0 mm in length, as the controls. The CMI IS-III Active^®^ S-Narrow (NeoBiotech Co., Seoul, Republic of Korea) and the CMI IS-III Active^®^ Narrow (NeoBiotech Co., Seoul, Republic of Korea) were used in the experimental groups; the implants were 3.2 mm and 3.5 mm in diameter, respectively; both experimental implants were 10.0 mm, 11.5 mm, or 13.0 mm in length (Table 1). Each patient received two implants in the anterior mandible with a provisional splinted prosthesis placed on the day of the implant surgery. The primary outcomes assessed were the one-year implant survival rate, success rate, and peri-implant marginal bone loss, as measured using periapical radiographs. Secondary outcomes included peri-implant soft tissue health, patient satisfaction, and esthetics evaluation.

### 2.2. Study Populations and Inclusion/Exclusion Criteria

In this study, the patients were recruited at the Department of Prosthodontics, School of Dentistry, Seoul National University, Seoul, Republic of Korea, between 11 March 2021 and 22 June 2023.

The inclusion criteria were as follows:

- 19 years of age or older
- Extractions and implant placement of at least two mandibular incisors required
- Healthy adjacent teeth periodontium
- Inter-implant distance ≥ 3 mm; teeth-implant distance ≥ 1.5 mm
- Sufficient amount of bone to obtain primary stability.

The exclusion criteria were as follows:

- Presence of acute infection in surgical sites
- Extraction of adjacent teeth required due to extensive periodontal disease
- Pregnancy or nursing
- Uncontrolled metabolic systemic diseases (hypertension, diabetes)
- Severe cardiovascular diseases, respiratory diseases, renal or liver diseases, digestive system diseases, blood system diseases, and neuropsychiatric diseases
- Hyperthyroidism or hypothyroidism
- Known history of drug allergy
- Severe depression or anxiety disorder
- History, or current abuse of, drug or alcohol abuse within a year
- Bisphosphonate medication within 4 months
- Heavy smoker
- Lack of occlusal stop.

The required sample size was calculated using the analysis of variance (fixed effects, omnibus, one-way) of parallel design using G*power 3.1.9.2 (University of Düsseldorf, Düsseldorf, Germany). The calculation was based on a previous randomized controlled clinical study by Al-Nawas et al. [18] on small-diameter bone-level implants. The random allocation ratio of the groups was 1:1:1 (control group–experimental group 1–experimental group 2). The estimated survival rates were 0.988, 0.99, and 0.975 for the control, experimental group 1, and experimental group 2, respectively. The standard deviation was set as 0.01. The alpha error probability was 0.05, and the statistical power was 0.80. The required number of implants in each group was nine; therefore, the required number of participants in each group was five. Considering the expected total dropout rates of 20%, 10% by patients and 10% from clinical study procedures, seven subjects were required in each group. Therefore, 21 patients were included in this study.

The patients were randomly allocated in a 1:1:1 ratio to control, experimental group 1, or experimental group 2, by one of the study coordinators shortly after they were qualified as study participants. The same study coordinator performed randomization, patient allocation, and enrollment. The randomization was performed using a computerized random number generator software (Excel 16.0, Microsoft, Redmond, Washington, WA, USA).

This study was conducted in accordance with the Declaration of Helsinki on experimentation with humans (World Medical Association, 2001). The study protocol was reviewed and approved by the Institutional Review Board of the Seoul National University Dental Hospital (IRB No. CDE21004). Written informed consent was obtained from all patients. This study was registered in the clinical registry (Cris, number: KCT0007300), and performed in compliance with CONSORT guidelines (S1 Checklist).

### 2.3. Treatment Procedures

#### 2.3.1. Virtual Planning, 3D Surgical Templates, Provisional prostheses

CBCT (Dinnova 3; HDXWILL, Seoul, Republic of Korea) and intraoral scans (Trios 4^®^; 3Shape, Copenhagen, Denmark) were performed in all patients who satisfied the inclusion criteria. Using Implant Studio^TM^ (3Shape, Copenhagen, Denmark), the two images were superimposed to evaluate bone dimensions and plan implant positions. The corresponding surgical templates were designed and produced using stereolithography (Stratasys, CA, USA). Implant planning and template designing were first roughly performed by dental technicians and then corrected and confirmed by the prosthodontist.

Fixed and removable provisional prostheses were prepared. Temporary resin shells and abutments were prepared for immediate provisionalization according to a protocol. A temporary denture was fabricated to deliver when the primary stability value did not meet the criteria for immediate provisionalization.

#### 2.3.2. Surgical Procedure

Each patient received two implants in the mandibular incisor area from the assigned group. Natural incisors were extracted, and implants were placed immediately in periodontally healthy sites. Implants were placed within six weeks following tooth extraction in cases of periodontally compromised sites.

**Figure 2:**
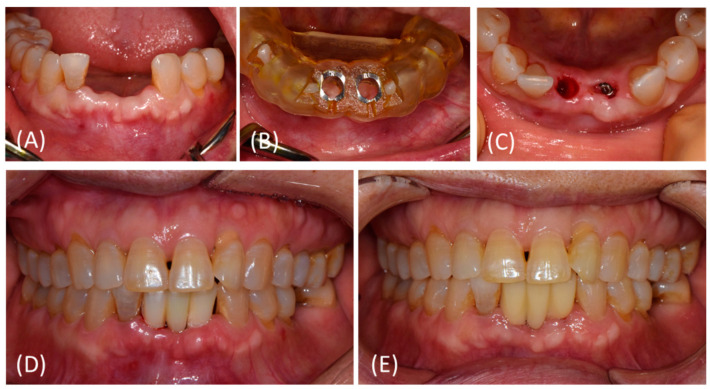
Representative photos of treatment procedures. (**A**–**C**) Implant placement at #32, 41 site using a surgical template without flap opening. The surgical template was removed after initial drilling. (**D**) Immediate provisionalization was performed on the day of surgery. (**E**) At 12 weeks after surgery, no pathologic sign was detected. Customized titanium abutments and zirconia bridge (screw- and cement-retained prosthesis) were delivered.

The intra-oral fit of the surgical template was evaluated. Local anesthesia was administered in the surgical area. Template-guided initial drilling was performed without elevating the flap. Subsequently, the surgical template was removed, and the flap was reflected minimally. After osteotomy and implant placement, bone grafting material was added if necessary. When the marginal gap was less than 2 mm, bone grafting was not performed. The implants were placed according to the manufacturer’s instructions (Figure 2). The insertion torque was measured by using a torque wrench. The torque values were adjusted to between 35 and 45 Ncm. When the insertion torque exceeded 45 Ncm, the implant was rotated in the reverse direction and re-tightened. The implant stability quotient (ISQ) was measured via resonance frequency analysis using an Osstell Mentor^®^ (Osstell AB, Göteborg, Sweden). An implant stability test (IST) was used to measure the implant stability using Anycheck (Neobiotech, Seoul, Republic of Korea). Implants with stability values < 65 were excluded from the study. Periapical radiographs were obtained on the day of surgery and at 4, 8, 12, 24, 36, and 48 weeks after surgery using a positioning jig for standardization.

After surgery, the patients received antibiotics (cefnidir, 100 mg three times daily), analgesics (acetaminophen, 650 mg as needed), and 0.1% chlorhexidine mouthwash for use three times daily for seven days.

#### 2.3.3. Prosthetic Procedure

On the day of the surgery, a provisional prosthesis was delivered. A fixed- and screw-type provisional implant bridge was placed when the primary stability was within an acceptable range. If an implant failed to meet the required values, the patient was excluded from the study, and a removable temporary denture was relined and delivered. For the patients who meet the implant stability criteria, temporary abutments were connected to the fixtures with minimum torque. The prefabricated temporary polymethyl methacrylate bridge was relined and adjusted. Access holes were made, and the temporary bridge was cemented using temporary cement. The holes were closed with a temporary filling material. An occlusal adjustment was performed so that during maximum intercuspation, a single 8 µm thick shim-stock could be withdrawn with resistance. The eccentric contacts were removed. The patients were instructed to avoid using their incisors.

Eight weeks after surgery (visit 7), a digital impression was taken for the fixtures with implant stability values ≥ 65 and with no pathologic sign. A scan body was connected, and the intraoral scan was performed using Trios 4^®^ (3Shape, Copenhagen, Denmark). The scan body for each implant type was as follows: IS OS Oral Scan Body; IS Oral Scan Body, Neobiotech, Seoul, Republic of Korea; CARES^®^ NC Mono Scan Body, Straumann, Basel, Switzerland. Customized titanium abutments and a monolithic zirconia bridge were designed using Implant Studio^TM^ (3Shape, Copenhagen, Denmark) and fabricated using a CAD/CAM system. Twelve weeks after the surgery (visit 8), implant stability was evaluated again using the Osstell and AnyCheck devices. A definitive screw and cement retained prostheses were placed, and occlusal adjustment was performed (Figure 2). The eccentric and protrusive contacts were eliminated. Panoramic and periapical radiographs were also obtained.

#### 2.3.4. Marginal Bone Loss Evaluation

**Figure 3:**
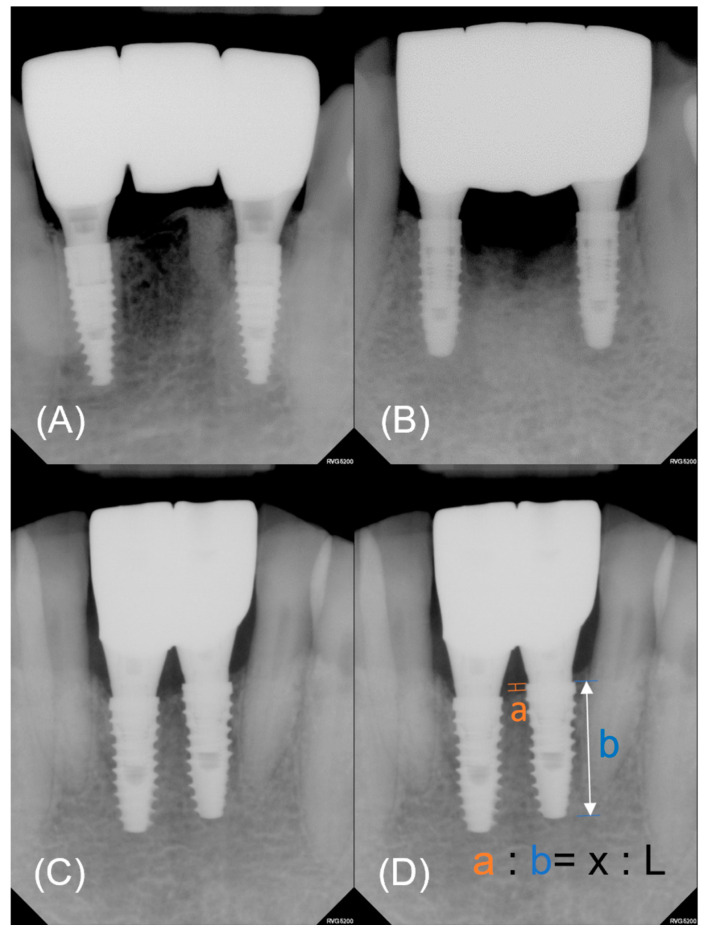
Representative periapical radiographs of each group. (**A**) Control. (**B**) Experimental group 1. (**C**) Experimental group 2. (**D**) The actual value of marginal bone loss (x) was calculated by converting the measured value (a) using the amplification ratio. The amplification ratio was obtained from the known length of a fixture (L) and measured length (b).

The primary outcome was peri-implant marginal bone loss (MBL). Periapical radiographs taken at 12, 24, and 48 weeks after surgery were used for the measurement. The amplification ratios of the radiographs were calculated using the known length of the implant from each manufacturer. Using the top of the fixture platform as a reference point, the distances from the bone crest were measured at the mesial and distal sides of the fixture. The actual values were calculated using the amplification ratios (Figure 3).

#### 2.3.5. Peri-Implant Soft Tissue Health, Patient Satisfaction, and Esthetic Score

At each follow-up visit from four weeks after surgery, the peri-implant soft tissue health was evaluated using the modified plaque index, modified sulcus bleeding index, pocket depth, and width of keratinized mucosa. The modified plaque indices and sulcus bleeding indices [19] were measured at six sites around the implant (mesiobuccal, midbuccal, distobuccal, mesiolingual, midlingual, and distolingual). The pocket depth was measured at four sites (buccal, lingual, mesial, and distal). The width of the keratinized mucosa was measured at three sites on the buccal gingiva (mesial, middle, and distal). For each parameter, average values were used.

A patient satisfaction survey was conducted using the visual analog scale (VAS) and the Oral Health Impact profile-14 (OHIP-14). The VAS was measured on a 0–100 mm scale for discomfort, time taken, satisfaction, and willingness to undergo the same procedure for impression making, surgery, and definitive prostheses. Average analgesic intake was also investigated. The OHIP-14 [20] was administered on the day of surgery and at 1 and 24 weeks after surgery (Figure 3).

The pink esthetic score and white esthetic score, as introduced by Fürhauser [21] and Belser [22], respectively, were performed at 12, 24, and 46 weeks after prosthesis delivery. The pink and white esthetic scores were assessed from the frontal side of the restorations in relation to the adjacent teeth. The parameters of the pink esthetic scores were as follows: (1) Mesial papilla filling. Score 0: absent; 2: incomplete; 3: complete. (2) Distal papillary filling. Score 0: absent; 2: incomplete; 3: complete. (3) Level of soft tissue margin. Score 0, major discrepancy > 2 mm; 1, minor discrepancy 1–2 mm; 2, no discrepancy. (4) Soft tissue color. Score 0: unnatural; 1: fairly natural; 2: natural. (5) Alveolar process contours. Score 0: obvious; 1: slight; 2: none. (6) Soft tissue color. Score 0: obvious difference; score 1: moderate difference; score 2: no difference. (7) Soft tissue texture. Score 0 = obvious difference; score 1 = moderate difference; score 2 = no difference [21]. The parameters in the white esthetic score were as follows: (1) tooth form, (2) tooth volume/outline, (3) color (hue and value), (4) surface texture, and (5) translucency. All parameters were assessed based on the following: score 0, major discrepancy; score 1, minor discrepancy; and score 2, no discrepancy [22].

#### 2.3.6. Follow-Up Visits and Implant Survival and Success

At 24, 36, and 48 weeks postoperatively, the patients were recalled for radiographic and intraoral examinations. Implant stability, peri-implant soft tissue health, and periapical radiographs were also evaluated. The clinical success criteria were as follows: (1) absence of consistent pain, foreign body sensation, or dysesthesia; (2) absence of recurrent peri-implantitis with pus discharge; (3) absence of implant fixture mobility; (4) absence of consistent radiolucency around the implant fixture; and (5) less than 5 mm of clinical periodontal pocket depth with bleeding [23]. In success rate calculation, implant “success” was defined as an implant that both fulfilled the clinical success criteria and exhibited implant stability values ≥ 65, as required by the study protocol. Implant “survival” was defined as an implant in situ at the end of the protocol, regardless of its health.

### 2.4. Statistical Analysis

Per-protocol analyses were used to compare the three groups. Intention to treatment and per-protocol analyses were used to compare the three groups. The χ^2^ test was used for categorical variables and the Kruskal–Wallis test was used for differences between groups using IBM SPSS Statistics (Version 26) (SPSS Inc., Chicago, IL, USA). The Mann–Whitney U test was performed to determine significant differences between pairs according to the Kruskal–Wallis test. The level of significance (*p* = 0.05) was adjusted using the Bonferroni correction. Two-way repeated-measures analyses of variance were performed after the verification of sphericity using the Huynh–Feldt method to evaluate the differences in the patterns of implant stability value changes over time. In order to describe the survival data, cumulative survival rate was illustrated using the Kaplan–Meier survival curve, and the log-rank test (Mantel–Cox test) was conducted to compare the survival rate for each group.

## 3. Results

### 3.1. Demographics

A total of 23 candidates were screened, out of which two were excluded based on the entry criteria. Eight patients were excluded before the definitive prosthesis delivery. One subject from the control group chose to withdraw from the study one month after the surgery. Two subjects were excluded because of low implant stability (<65). Five subjects experienced at least one fixture failure (Figure 1). The age range of the 18 patients (8 males and 10 females) fell between 55 and 78, with an average age of 65 ± 6.16 years. There were no statistically significant differences observed in terms of age or sex of the patients (*p* > 0.05, Table 2). A total of 9 implants were placed in the central mandibular area, while the remaining 27 were placed in the lateral mandibular incisor areas. The prosthetic units ranged from two to four. Comprehensive details and breakdown of the failed implants are shown in Table 3.

### 3.2. Survival Rate and Success Rate

With the exception of one patient, all excluded patients had only a single implant, which did not satisfy the criteria for success. One patient experienced failure of both fixtures (Table 3). According to the protocol, patients with any unsuccessful implants were excluded from further participation, and the tracking of the remaining implants was discontinued. The failed implants were removed and replaced, with the delivery of definitive prostheses taking place three months post-surgery.

**Figure 4:**
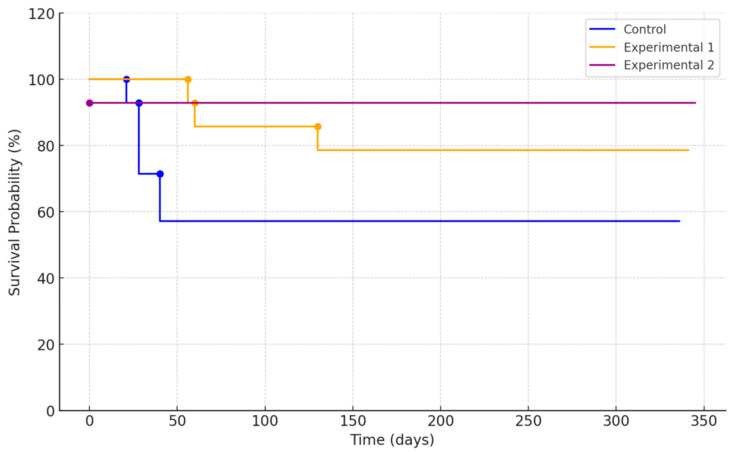
Kaplan–Meier survival curve of each implant group.

For the patients who diligently completed all prescribed clinical protocols, the implant success rate was 100%. Throughout the one-year observation period, the percentages of patients lost due to failure to meet the requisite criteria were 50.0%, 42.9%, and 14.3% in the control, experimental group 1, and experimental group 2, respectively. Regarding implant survival rates, the percentage of implants in situ at the conclusion of the one-year clinical study period were 66.7%, 85.7%, and 100% in the control, experimental group 1, and experimental group 2, respectively. Regarding implant survival rates, the total number of followed-up implants were 12, 14, and 14 for the control, experimental group 1, and experimental group 2, respectively. The number of surviving implants, or the fixtures that were not removed after surgery, were 8, 12, and 14 for the control, experimental group 1, and experimental group 2, respectively. Among the “failed implants” described in Table 3, those with low stability values were not removed unless they showed pathological signs. Therefore, the percentages of implants in situ at the conclusion of the one-year clinical study period were 66.7%, 85.7%, and 100% in the control, experimental group 1, and experimental group 2, respectively. The Kaplan–Meier survival analysis curve is shown in Figure 4. When performing the log-rank test, no statistically significant difference was observed in survival time among the three groups (*p* = 0.999).

### 3.3. Marginal Bone Loss

MBL was analyzed in patients with periapical radiographs available at 12, 24, and 48 weeks following implant placement. Minimal peri-implant bone resorption was observed consistently over the observation period. The MBL on the distal side tended to be smaller compared to the mesial side. There were no statistically significant differences between the groups (Table 4).

### 3.4. Implant Stability Comparison

**Figure 5:**
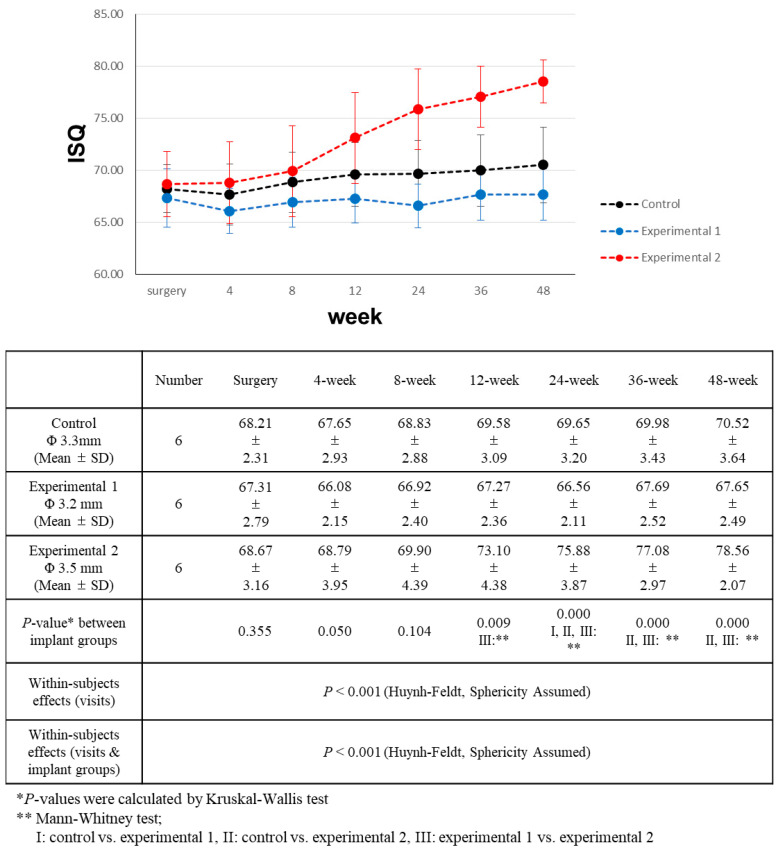
Comparison of implant stability quotient (ISQ) value changes over a 48-week period.

**Figure 6:**
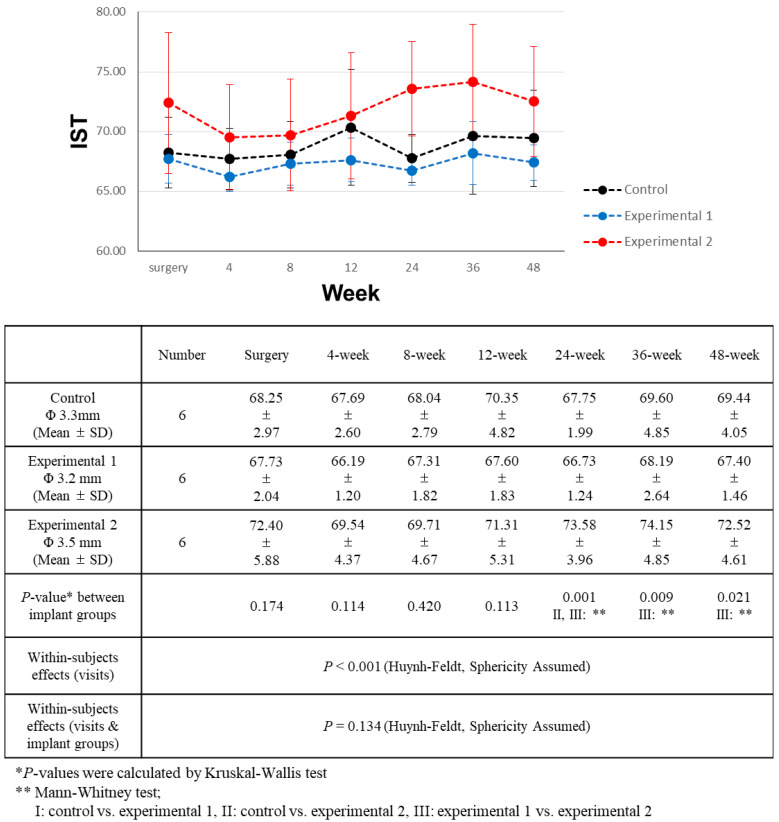
Comparison of implant stability test (IST) value changes over a 48-week period.

The primary stability was assessed using the peak insertion torque, and concurrent implant stability values were recorded at the time of implant placement. Although experimental group 1 had slightly greater insertion torque, ISQ, and IST values, statistical analysis revealed that these differences lacked significance. All patients except one (experimental group 2) showed acceptable stability during the surgical procedure (Table 5). Subsequent ISQ and IST measurements were taken at intervals of 4, 8, 12, 24, 36, and 48 weeks following the surgery. The measured ISQ and IST values are shown in Figure 5 and Figure 6, respectively.

During the entirety of the follow-up period, experimental group 2 consistently showed higher ISQ values than the other groups, with this difference becoming more pronounced over time. On the day of surgery, as well as at 4 and 8 weeks post-surgery, the differences were not statistically significant. However, at the 12-week mark post-surgery, experimental group 2 had significantly higher ISQ values than experimental group 1. Subsequently, at the 24-week post-surgery point, the ISQ value of experimental group 2 was significantly higher than that of both the control and experimental group 1. This trend persisted during the 36-week and 48-week measurements after surgery.

All three groups exhibited increasing ISQ values throughout the observation period. For experimental group 2 (CMI IS-III Active^®^ Narrow, Φ 3.5 mm), the ISQ values showed a significantly positive correlation with the number of visits. However, for the control group (BLT NC SLActive^®^, Φ 3.3 mm), the upward trend of ISQ values over time demonstrated a weaker correlation (Table 5). In experimental group 1 (CMI IS-III Active^®^ S-Narrow, Φ 3.2 mm), ISQ values exhibited minimal variation over the follow-up period. Four weeks after surgery, a slight decrease in stability was observed in the control and experimental group 1.

Overall, the observed IST values exhibited variations, although these differences were modest. Across all groups, there was a noticeable upward trend in IST values. However, these values did not exhibit a meaningful correlation with time elapsed since the surgery (Table 6). Group 2 consistently displayed higher IST values, albeit with minimal differences. Significantly discernible differences emerged only starting from the 12-week mark after surgery. At the 24-week juncture post-surgery, experimental group 2 showed significantly higher IST values than both experimental group 1 and the control group. This divergence persisted at the 36- and 48week post-surgery assessments, where the IST values of experimental group 2 remained significantly higher than those of experimental group 1.

### 3.5. Soft Tissue Health Parameter

The condition of the soft tissue surrounding the implants was evaluated using the modified plaque index, modified sulcus bleeding index, pocket depth, and width of the keratinized mucosa. At 48 weeks post-surgery, all remaining implants showed healthy peri-implant soft tissue conditions and fulfilled the established success criteria. There were no statistical differences among the groups in terms of plaque index, sulcus bleeding index, pocket depth, and width of the keratinized mucosa (Table 7).

### 3.6. Pink and White Esthetic Score

At 48 weeks after the delivery of the definitive prosthesis, the esthetics of both the adjacent tissue and the prosthesis were evaluated using the pink esthetic score and white esthetic score, respectively. The mean total scores were then compared. Both pink and white esthetic score values ranked highest in experimental group 2, followed by experimental group 1 and the control group. However, there was no statistically significant difference among the three groups (Table 8).

### 3.7. Patient Satisfaction

A patient satisfaction survey was performed using the VAS at various stages: after an oral scan and alginate impression, surgery, one week post-surgery, three weeks post-surgery, definitive prosthesis delivery, and three months after the delivery of definitive prosthesis (Figure 3). This survey assessed discomfort and time taken for each conventional alginate impression and oral scan. No significant differences were observed between the two impression techniques. Postoperative pain, swelling, surgery time, surgery satisfaction, and intention to repeat the same operation were assessed post-surgery, definitive prosthesis delivery, and three months after definitive prosthesis delivery. The analysis showed no significant differences in patient satisfaction between the groups at any time point. Postoperative pain and swelling improved at one and three weeks post-surgery. Three months after definitive prosthesis delivery, there was little difference in pain, swelling, time taken, or willingness to repeat the same procedure; however, satisfaction with the prosthesis itself showed a decrease at this juncture.

To further assess general discomfort stemming from oral problems, the OHIP-14 survey was administered before and after surgery and at the three month milestone after the delivery of the definitive prosthesis. There were no significant differences among the three groups at any time point. Three months after definitive prosthesis delivery, the patients showed improvements in most items and the largest improvement in pronunciation compared to before implant placement.

## 4. Discussion

This randomized controlled clinical trial sought to assess the one-year clinical performance of three different NDIs in the mandibular incisor area: BLT NC SLActive^®^ (Straumann, Basel, Switzerland), CMI IS-III Active^®^ S-Narrow (NeoBiotech Co., Seoul, Republic of Korea), and CMI IS-III Active^®^ Narrow (NeoBiotech Co., Seoul, Republic of Korea). In total, eight patients discontinued the procedure: one patient voluntarily dropped out, two patients showed low implant stability value < 65, and five patients had at least one fixture failure before definitive prosthesis delivery.

Two patients, one from each experimental group, were excluded due to their low ISQ and IST values of less than 65. The ISQ value, obtained using the Osstell device, is based on the resonance frequency analysis principle. This method involves applying oscillations to the implant, measuring the integrated surface between the implant and the bone. This non-invasive and straightforward technique is widely accepted and not sensitive to variations in execution [24]. The AnyCheck device measures the modified damping capacity of implants by tapping the healing abutment connected to the fixture. The converted AnyCheck value reflects implant stability. Both devices’ assessment criteria correlate closely, with recommended values of 65 or higher for successful loading. Given their shared basis in osseointegration assessment, smaller fixture surface area NDIs inherently face a disadvantage. Kim [25] and Guler [26] reported increased ISQ values in larger-diameter implants. In instances of immediate or early implant placement, fixture stability primarily depends on engagement with the lower part of the bone within the extraction socket. However, due to the dimensional disparities between the extraction socket and the fixture, a marginal gap is formed. Compared to regular- or wide-diameter implants, NDI implants have a smaller bone-engaged area and a greater marginal gap, and the measured stability value may be smaller than the actual value.

Only experimental group 2 (Φ 3.5 mm) showed a significant increase in ISQ values over the one-year observation period. On the other hand, the ISQ values for the experimental group 1 (Φ 3.2 mm) and control (Φ 3.3 mm) in this study were maintained at the values measured at the time of implant surgery. This outcome can be attributed to a plausible direct correlation between ISQ values and bone-to-implant contact area [27,28,29]. In this context, it is recommended to use NDI with a wide diameter in patients with excessive occlusal force from a clinical perspective.

This prospective study evaluated the survival and various clinical and radiographic parameters of three different types of NDIs inserted in the anterior mandible of partially edentulous patients. To the best of our knowledge, there was a lack of direct comparable studies available for reference. Existing literature predominantly addresses NDIs placed across various oral regions or in the posterior sections of both jaws. When discussions pertain specifically to NDIs in the anterior mandible, they often pertain to supporting overdentures in either fully edentulous patients or those requiring a single prosthesis. Although many studies have reported survival rates, success rates, and peri-implant soft tissue parameters, limited research has explored changes in implant stability over time. For studies that specified the dimensions of the NDIs, the observed sizes exhibited variability, yet a diameter of 3.3 mm was frequently encountered.

Most studies on the clinical results of NDIs supporting fixed prostheses have observed higher survival rates than those in the present study. A recent one-year prospective study reported a survival rate of 96.9% for 3.3 mm NDIs within the anterior jaws [7], in contrast to the implant survival rate of 66.7% observed in this study. Retrospective analyses of 3.3 mm NDIs have yielded survival and success rates of 90.0–100% when placed in the anterior mandible [30,31]. Several prospective studies and meta-analyses have the NDIs placed in the posterior jaws, demonstrating high survival rates ranging from 93.8–100% [8,11,32,33,34,35]. Similar survival rates have been reported for NDIs with diameters below 3.3 mm [8,34]. A meta-analysis by Ortega-Oller [36] reported a significantly lower survival rate (75%) for implants with diameters below 3.3 mm, as opposed to 87% for diameters equal to or greater than 3.3 mm. Although that meta-analysis included the posterior region, the findings align with those of the present study.

Experimental group 2 (Φ 3.5 mm) showed the highest survival and success rates, followed by experimental group 1 (Φ 3.2 mm) and the control (Φ 3.3 mm). Excluding voluntary dropout and insufficient ISQ, all instances of fixture failures were observed in the control and experimental group 1. Failed implants were successfully replaced with larger-diameter or longer implants. Our study suggests that a critical threshold exists for implant surface area to achieve adequate osseointegration for immediate loading. Osseointegration failure was observed only in groups with 3.2 and 3.3 mm diameters, which is concurrent with previous meta-analysis [36]. However, our study solely categorized implants by diameter, overlooking potential complexities in surface area. This suggests that diameter may have a greater influence on osseointegration than surface area, but further research controlling implant dimensions is necessary for clarity. Interestingly, primary stability appears less influenced by diameter or implant dimensions. Notably, among failed implants, those lacking adequate primary stability were from experimental group 2.

In contrast to our study, a successful clinical outcome was shown using NDI in the anterior mandibular region in the previous study [7,30,31,37]. This discrepancy might be due to the healing state of the ridge. In the previous study, most of the NDIs were placed in the completely healed ridge sites, whereas NDIs in our study were placed in the extraction socket or incompletely healed ridge sites. Therefore, successful immediate loading following immediate or early implant placement might be guaranteed using 3.5 mm diameter implants. Further studies are essential to accurately determine the surface area dimensions required for successful osseointegration.

It is important to note that these implants were constantly exposed to trauma from tongue and lip movements. Functional movements during speaking, swallowing, and facial expressions introduce forces that can stress anterior prostheses. Removal torque from repeated ISQ and IST measurements should also be considered in cases of early osseointegration failure. Each ISQ measurement required the removal of the healing abutment and the subsequent tightening of the SmartPeg. The IST measurement involves mechanical tapping on a healing abutment.

Following the delivery of the definitive prosthesis, no patients experienced fixture failure. The stiffness of the definitive restoration might have contributed to stabilizing the implants and reinforcing a stronger splinting effect. This splinting effect holds a significant influence on implant stability. The splinting of multiple implants effectively reduces stress on adjacent bone tissue by distributing the force among the splinted implants [38,39,40]. Overloaded stress can detrimentally affect bone tissue, influencing bone remodeling, marginal bone loss, and osseointegration failure [41,42]. This suggests that the splinting effect likely countered the stress on the fixture and contributed to the implant success in this study. While it is plausible that an increase in the number of implant-supported fixed prostheses could adversely affect the outcome, leading to the strain on the same number of implants to withstand increased stress, our study’s limited sample size did not yield a substantial increase in the failure rate.

A systematic review demonstrated that the MBL of internal bone level implants ranged between 0.12 mm and 0.87 mm [43]. After one year post-surgery, all monitored implants showed approximately 0.4 mm of MBL, which coincides with the result of the systematic review. It be noted that while MBL measurements were conducted in a mesiodistal direction, bone resorption at the recipient site predominantly occurred buccally.

A fully digital approach was used for the planning, surgical guide fabrication, and creation of provisional prostheses. However, due to the lack of buccal bone dimensions, the resultant prosthesis fell short of being mechanically or esthetically ideal. The implant position was planned so that the fixture resided within the remaining bone structure, positioned lingually to the ideal location for the prostheses. The prosthesis was extended buccally, resulting in a cantilever buccally positioned unit. This lingually positioned implant fixture placement, albeit maximizing remaining bone, could encroach upon tongue space, causing discomfort to the patient and pronunciation challenges. However, there was no significant difference in esthetic scores among the three groups, since the contour of a prosthesis is determined largely by the implant position rather than the implant size. A comprehensive evaluation of the surgical site and a detailed consideration of the implant position based on the bone dimension were performed during the planning protocol. Given the limitations in bone dimensions, such planning is not only recommended but potentially essential. Guided osteotomy, for instance, enables accurate placement as planned [44]. The implant failure rate was reduced in computer-assisted implant surgery compared to freehand methods [31].

Despite the expected advantages of oral scanning, there was no difference in patient discomfort or time taken between conventional impression-taking and digital oral scanning. Digital oral scanning eliminates the need for impression materials, bypassing the requirement for working and setting times for impression materials and stone. This could ostensibly streamline the entire model-making process. However, the familiarity of both patients and clinicians with conventional impression-taking might have rendered it more acceptable for patients and more comfortable for clinicians. The result may have differed if less flexible rubber impression materials were used instead of alginate in our study. Given its hardness and size, the use of a plastic intraoral scanner can cause discomfort when scanning the entire arch. Additionally, the mandibular anterior region typically has good accessibility for dental procedures, and any differences between the two impression-taking procedures may have been obscured.

Patient satisfaction with definitive prosthesis delivery decreased after three months. The initial advantage of promptly restoring the anterior teeth might have inadvertently led to this outcome. Immediate provisionalization seemed to alleviate the patients’ fear of missing front teeth and the ensuing social perceptions. The relief and contentment from provisional prostheses, symbolizing the recovery of front teeth, appeared to overshadow the transition to definitive prostheses. The available bone volume was not ideal for implant placement in multiple patients. The primary focus during implant planning was on sites with optimal bone mass, which did not always coincide with the original tooth position, often relegating esthetic concerns to a secondary role. Vertical bone loss results in a lengthened crown. However, this procedure has clinical implications for patients in maintaining social activities with immediate restoration of the front teeth.

Despite the limitation of a small sample size, the outcomes from CMI IS-III Active^®^ Narrow implants (NeoBiotech Co., Seoul, Republic of Korea) showed the best clinical results. CMI IS-III Active^®^ S-Narrow (NeoBiotech Co., Seoul, Republic of Korea) implants showed higher success and survival rates than control implants (Straumann, Basel, Switzerland), despite the lack of statistical significance. Conducting the current study with NDIs in the anterior mandible proved to be a challenging endeavor. The reduced surface area of the fixtures adversely affected early osseointegration with the surrounding bone, further compounded by exposure to multiple sources of trauma. Therefore, obtaining the maximum implant–bone contact area is critical, especially during immediate loading. Augmenting the implant surface area can be achieved either through using a larger diameter or an extended length. The findings from this study suggest that a diameter exceeding 3.3 mm is required for stable osseointegration during immediate placement and loading. Olate [45] observed that short implants contributed significantly to increased early failure rates. Al-Nawas [7] reported a higher survival rate for longer implants. This additional length confers a larger osseointegration surface area, compensating for the diminished surface area due to the smaller diameter. A delayed-loading protocol is recommended when sufficient implant stability is not achieved.

The limitations of the present study include its relatively modest sample size. The one-year observation period was also relatively short to make definitive statements about the clinical outcomes. Also, this study was single-center and single-blinded. The dentists were aware of the implant products during surgery and clinical examinations, which could have influenced the results.

## 5. Conclusions

This prospective study evaluated one-year clinical and radiographic outcomes of three different types of narrow-diameter implants (BLT NC SLActive^®^ (Straumann, Basel, Switzerland), CMI IS-III Active^®^ S-Narrow (NeoBiotech Co., Seoul, Republic of Korea), and CMI IS-III Active^®^ Narrow (NeoBiotech Co., Seoul, Republic of Korea)) in the mandibular incisor region with immediate loading. The failure rates exhibited an upward trend for implants with smaller diameters (Φ 3.2 mm, 3.3 mm). Within the limitations of this study, the reduced implant dimensions may yield a critical difference in the implant–bone interface area for successful osseointegration. For immediate loading, a minimum diameter of 3.5 mm appears necessary.

## Figures and Tables

**Table 1:** Characteristics of the implant systems used in this study.

	Control Group	Experimental 1	Experimental 2
	Straumann^®^ BLT NC, SLActive^®^	NeoBiotech CMI IS-III Active^®^ S-Narrow	NeoBiotech CMI IS-III Active^®^ Narrow
Diameter	Φ 3.3 mm	Φ 3.2 mm	Φ 3.5 mm
Body Shape	Tapered Body	Straight Body	Straight Body
Pitch Height	0.8 mm	0.9 mm	0.9 mm
Thread Depth	0.3 mm	0.25 mm	0.35 mm
Implant-Abutment Interface	Internal Conical (15°)	Internal Conical (11°, 2.1 hex)	Internal Conical (11°, 2.1 hex)
Surface Treatment	SLA surface	SLA surface	SLA surface
Micro Threads	None	None	None

**Table 2:** Demographic data of the participants.

	Variables	Control (Straumann BLT^®^ Φ 3.3 mm NC, SLActive^®^)	Experimental 1 (NeoBiotech IS-III Active^®^ S-Narrow)	Experimental 2 (NeoBiotech IS-III Active^®^ Narrow)	*p*-Value *
Subject based (N = 18)	Participant number	6	6	6	-
	Age (Mean ± SD)	61.17 ± 4.98	67.50 ± 5.88	66.33 ± 5.62	
	20–60	3	2	1	0.532
	Over 60	3	4	5	0.378
	Sex Male/Female	4/2	1/5	3/3	0.226
Implant based (N = 36)	Implant number	12	12	12	-
	Mandibular central incisors (Left/right)	0/0	2/3	1/3	
	Mandibular lateral incisors (Left/right)	6/6	3/4	5/3	
	Prostheses unit number (2/3/4)	0/0/6	2/2/2	1/2/3	
	Implant type (number of implants)	Φ 3.3 × 10 mm (9)	Φ 3.2 × 10 mm (8)	Φ 3.5 × 10 mm (8)	
		Φ 3.3 × 12 mm (3)	Φ 3.2 × 11.5 mm (4)	Φ 3.5 × 11.5 mm (4)	
Bone graft	Number (application/implants)	5/12	7/12	4/12	

* *p*-values were calculated by Kruskal–Wallis test.

**Table 3:** Description of failed implants.

Implant Type	Number of Failed Patients/Implants	Time Point	Implant Position	Implant Size	Bone Graft	Failure Mode	Cause of Failure
Control * (BLT Φ 3.3 mm NC, SLActive^®^)	3/4	Visit 5	#32	Φ 3.3 × 10 mm	Yes	Fixture failure	Severe peri-implantitis
		Visit 6	#32	Φ 3.3 × 10 mm	No	Fixture failure	Osseointegration failure (peri-implant radiolucency)
		Visit 7	#32 **	Φ 3.3 × 12 mm	No	Fixture failure	Osseointegration failure (mobility without peri-implant radiolucency)
		Visit 7	#42 **	Φ 3.3 × 12 mm	No	Fixture failure	
Experimental 1 (CMI IS-III Active ^®^ S-Narrow Φ 3.2 mm)	3/3	Visit 5	#42	Φ 3.2 × 10 mm	Yes	Fixture failure	Use of hard food reported
		Visit 7	#32	Φ 3.2 × 10 mm	Yes	ISQ or IST < 65	
		Visit 7	#31	Φ 3.2 × 10 mm	No	Fixture failure	Osseointegration failure (peri-implant radiolucency)
Experimental 2 (CMI IS-III Active ^®^ Narrow Φ 3.5 mm)	1/1	Visit 3 (Surgery)	#42	Φ 3.5 × 10 mm	No	ISQ or IST < 65	

* One voluntary drop-out patient was not included. ** Implants failed from one patient.

**Table 4:** Comparison of marginal bone loss between the control and experimental groups.

		Control	Experimental 1	Experimental 2	
Patient Number		3	4	6	
Duration	Area	Mean ± SD (mm)	Mean ± SD (mm)	Mean ± SD (mm)	*p*-Value *
12 week follow up	Mesial	0.00 ± 0.00	0.59 ± 0.59	0.71 ± 0.73	0.055
	Distal	0.19 ± 0.31	0.02 ± 0.05	0.25 ± 0.43	0.592
	Avg.	0.10 ± 0.15	0.30 ± 0.31	0.48 ± 0.50	0.224
24 week follow up	Mesial	0.20 ± 0.44	0.65 ± 0.41	0.68 ± 0.68	0.185
	Distal	0.33 ± 0.37	0.13 ± 0.36	0.17 ± 0.35	0.384
	Avg.	0.26 ± 0.23	0.39 ± 0.17	0.42 ± 0.40	0.684
48 week follow up	Mesial	0.32 ± 0.48	0.74 ± 0.39	0.64 ± 0.63	0.309
	Distal	0.43 ± 0.32	0.13 ± 0.33	0.10 ± 0.26	0.078
	Avg.	0.38 ± 0.36	0.43 ± 0.30	0.37 ± 0.37	0.749

* *p*-values were calculated by Kruskal–Wallis test, Mann–Whitney test.

**Table 5:** Comparison of primary stability at the surgery.

	Control	Experimental I	Experimental II	*p*-Value *
Participant number	6	6	6	-
Insertion torque (Ncm)(Mean ± SD)	37.92 ± 2.47	41.25 ± 3.61	37.92 ± 3.80	0.048
ISQ at surgery (Mean ± SD)	67.32 ± 2.79	68.67 ± 3.16	68.21 ± 2.31	0.355
IST at surgery (Mean ± SD)	67.72 ± 2.04	73.40 ± 5.88	68.25 ± 2.97	0.174

* *p*-values were calculated by Kruskal–Wallis test, Mann–Whitney test.

**Table 6:** Correlation index of ISQ and IST values vs. visits.

		Control	Experimental 1	Experimental 2
ISQ	Pearson Correlation Coefficient	0.274 *	0.111	0.709 **
	R^2^	0.075	0.012	0.503
IST	Pearson Correlation Coefficient	0.136	0.090	0.188
	R^2^	0.019	0.008	0.035

* *p* < 0.05, ** *p* < 0.01.

**Table 7:** Comparison of peri-implant soft tissue parameters between the control and experimental groups at 48-week follow-up.

	Control	Experimental 1	Experimental 2	
Participant number	4	5	6	
Parameters	Mean ± SD (mm)	Mean ± SD (mm)	Mean ± SD (mm)	*p*-value *
Modified plaque index	1.15 ± 0.87	1.05 ± 0.54	0.90 ± 0.58	0.113
Modified sulcus bleeding index	0.75 ± 0.63	0.63 ± 0.68	0.68 ± 0.57	0.540
Pocket Depth (mm)	3.53 ± 0.49	3.55 ± 0.43	3.35 ± 0.75	0.832
Width of keratinized mucosa (mm)	4.00 ± 0.50	4.40 ± 1.11	4.08 ± 1.38	0.750

* *p*-values were calculated by Kruskal–Wallis test.

**Table 8:** Mean pink esthetic score and white esthetic score at 48 weeks after surgery.

	PES (Maximum 14)	WES (Maximum 10)
	Mean ± SD	Mean ± SD
Control	8.00 ± 0.82	3.67 ± 0.47
Experimental 1	9.25 ± 2.86	5.50 ± 0.50
Experimental 2	10.50 ± 1.12	7.17 ± 2.54

## Data Availability

Data are contained within the article.

## References

[1] Couso-Queiruga E., Ahmad U., Elgendy H., Barwacz C., González-Martín O., Avila-Ortiz G. (2021). Characterization of Extraction Sockets by Indirect Digital Root Analysis. Int. J. Periodontics Restor. Dent..

[2] Chappuis V., Araújo M.G., Buser D. (2017). Clinical relevance of dimensional bone and soft tissue alterations post-extraction in esthetic sites. Periodontology 2000.

[3] Chiapasco M., Zaniboni M. (2009). Clinical outcomes of GBR procedures to correct peri-implant dehiscences and fenestrations: A systematic review. Clin. Oral Implants Res..

[4] Schiegnitz E., Al-Nawas B. (2018). Narrow-diameter implants: A systematic review and meta-analysis. Clin. Oral Implants Res..

[5] Davarpanah M., Martinez H., Tecucianu J.F., Celletti R., Lazzara R. (2000). Small-diameter implants: Indications and contraindications. J. Esthet. Restor. Dent..

[6] Degidi M., Piattelli A., Carinci F. (2008). Clinical outcome of narrow diameter implants: A retrospective study of 510 implants. J. Periodontol..

[7] Al-Nawas B., Domagala P., Fragola G., Freiberger P., Ortiz-Vigón A., Rousseau P., Tondela J. (2015). A prospective noninterventional study to evaluate survival and success of reduced diameter implants made from titanium-zirconium alloy. J. Oral Implantol..

[8] Klein M.O., Schiegnitz E., Al-Nawas B. (2014). Systematic review on success of narrow-diameter dental implants. Int. J. Oral Maxillofac. Implants.

[9] Bidra A.S., Almas K. (2013). Mini implants for definitive prosthodontic treatment: A systematic review. J. Prosthet. Dent..

[10] Vigolo P., Givani A., Majzoub Z., Oordioli G. (2004). Clinical evaluation of small-diameter implants in single-tooth and multiple-implant restorations: A 7-year retrospective study. Int. J. Oral Maxillofac. Implants.

[11] Alrabiah M. (2019). Comparison of survival rate and crestal bone loss of narrow diameter dental implants versus regular dental implants: A systematic review and meta-analysis. J. Investig. Clin. Dent..

[12] Cruz R., Lemos C., de Batista V., Yogui F., Oliveira H., Verri F. (2021). Narrow-diameter implants versus regular-diameter implants for rehabilitation of the anterior region: A systematic review and meta-analysis. Int. J. Oral Maxillofac. Surg..

[13] Ganeles J., Zöllner A., Jackowski J., Ten Bruggenkate C., Beagle J., Guerra F. (2008). Immediate and early loading of Straumann implants with a chemically modified surface (SLActive) in the posterior mandible and maxilla: 1-year results from a prospective multicenter study. Clin. Oral Implants Res..

[14] Ioannidou E., Doufexi A. (2005). Does loading time affect implant survival? A meta-analysis of 1266 implants. J. Periodontol..

[15] Ye M., Liu W., Cheng S., Yan L. (2022). Immediate vs conventional loading of mandibular overdentures: A comprehensive systematic review and meta-analysis of randomized controlled trials. J. Oral Implantol..

[16] Khzam N., Arora H., Kim P., Fisher A., Mattheos N., Ivanovski S. (2015). Systematic review of soft tissue alterations and esthetic outcomes following immediate implant placement and restoration of single implants in the anterior maxilla. J. Periodontol..

[17] Glauser R., Zembic A., Hämmerle C.H. (2006). A systematic review of marginal soft tissue at implants subjected to immediate loading or immediate restoration. Clin. Oral Implants Res..

[18] Al-Nawas B., Brägger U., Meijer H.J., Naert I., Persson R., Perucchi A., Quirynen M., Raghoebar G.M., Reichert T.E., Romeo E. (2012). A double-blind randomized controlled trial (rct) of titanium-13zirconium versus titanium grade iv small-diameter bone level implants in edentulous mandibles–results from a 1-year observation period. Clin. Implant Dent. Relat. Res..

[19] Mombelli A., Van Oosten M., Schürch E., Lang N. (1987). The microbiota associated with successful or failing osseointegrated titanium implants. Oral Microbiol. Immunol..

[20] Slade G.D. (1997). Derivation and validation of a short-form oral health impact profile. Community Dent. Oral Epidemiol..

[21] Fürhauser R., Florescu D., Benesch T., Haas R., Mailath G., Watzek G. (2005). Evaluation of soft tissue around single-tooth implant crowns: The pink esthetic score. Clin. Oral Implants Res..

[22] Belser U.C., Grütter L., Vailati F., Bornstein M.M., Weber H.P., Buser D. (2009). Outcome evaluation of early placed maxillary anterior single-tooth implants using objective esthetic criteria: A cross-sectional, retrospective study in 45 patients with a 2-to 4-year follow-up using pink and white esthetic scores. J. Periodontol..

[23] Karoussis I.K., Salvi G.E., Heitz-Mayfield L.J., Brägger U., Hämmerle C.H., Lang N.P. (2003). Long-term implant prognosis in patients with and without a history of chronic periodontitis: A 10-year prospective cohort study of the ITI^®^ Dental Implant System. Clin. Oral Implants Res..

[24] Kahraman S., Bal B., Asar N., Turkyilmaz I., Tözüm T. (2009). Clinical study on the insertion torque and wireless resonance frequency analysis in the assessment of torque capacity and stability of self-tapping dental implants. J. Oral Rehabil..

[25] Kim H.-J., Kim Y.-K., Joo J.-Y., Lee J.-Y. (2017). A resonance frequency analysis of sandblasted and acid-etched implants with different diameters: A prospective clinical study during the initial healing period. J. Periodontal Implant Sci..

[26] Guler A.U., Sumer M., Duran I., Sandikci E.O., Telcioglu N.T. (2013). Resonance frequency analysis of 208 Straumann dental implants during the healing period. J. Oral Implantol..

[27] Scarano A., Degidi M., Iezzi G., Petrone G., Piattelli A. (2006). Correlation between implant stability quotient and bone-implant contact: A retrospective histological and histomorphometrical study of seven titanium implants retrieved from humans. Clin. Implant Dent. Relat. Res..

[28] Nkenke E., Hahn M., Weinzierl K., Radespiel-Tröger M., Neukam F.W., Engelke K. (2003). Implant stability and histomorphometry: A correlation study in human cadavers using stepped cylinder implants. Clin. Oral Implants Res..

[29] Huang H.-L., Tsai M.-T., Su K.-C., Li Y.-F., Hsu J.-T., Chang C.-H., Fuh L.-J., Wu A.Y.-J. (2013). Relation between initial implant stability quotient and bone-implant contact percentage: An in vitro model study. Oral Surg. Oral Med. Oral Pathol. Oral Radiol..

[30] Romeo E., Lops D., Amorfini L., Chiapasco M., Ghisolfi M., Vogel G. (2006). Clinical and radiographic evaluation of small-diameter (3.3-mm) implants followed for 1–7 years: A longitudinal study. Clin. Oral Implants Res..

[31] Pedrinaci I., Sun T.C., Sanz M., Sanz-Esporrin J., Hamilton A., Gallucci G.O. (2023). Implant survival in the anterior mandible: A retrospective cohort study. Clin. Oral Implants Res..

[32] Lee J.S., Kim H.M., Kim C.S., Choi S.H., Chai J.K., Jung U.W. (2013). Long-term retrospective study of narrow implants for fixed dental prostheses. Clin. Oral Implants Res..

[33] Lambert F.E., Lecloux G., Grenade C., Bouhy A., Lamy M., Rompen E.H. (2015). Less invasive surgical procedures using narrow-diameter implants: A prospective study in 20 consecutive patients. J. Oral Implantol..

[34] Grandi T., Svezia L., Grandi G. (2017). Narrow implants (2.75 and 3.25 mm diameter) supporting a fixed splinted prostheses in posterior regions of mandible: One-year results from a prospective cohort study. Int. J. Implant Dent..

[35] Blanes R.J., Bernard J.P., Blanes Z.M., Belser U.C. (2007). A 10-year prospective study of ITI dental implants placed in the posterior region. I: Clinical and radiographic results. Clin. Oral Implants Res..

[36] Ortega-Oller I., Suárez F., Galindo-Moreno P., Torrecillas-Martínez L., Monje A., Catena A., Wang H.L. (2014). The influence of implant diameter on its survival: A meta-analysis based on prospective clinical trials. J. Periodontol..

[37] Ćorić A., Kovačić I., Kiršić S.P., Čelebić A. (2022). Are Mini Dental Implants Suitable for Support of Crowns or Small Bridges in the Mandibular Incisor Region? A 5-year Longitudinal Study. J. Oral Maxillofac. Surg..

[38] Bergkvist G., Simonsson K., Rydberg K., Johansson F., Dérand T. (2008). A finite element analysis of stress distribution in bone tissue surrounding uncoupled or splinted dental implants. Clin. Implant Dent. Relat. Res..

[39] Wang T.-M., Leu L.-J., Wang J.-S., Lin L.-D. (2002). Effects of prosthesis materials and prosthesis splinting on peri-implant bone stress around implants in poor-quality bone: A numeric analysis. Int. J. Oral Maxillofac. Implants.

[40] Guichet D.L., Yoshinobu D., Caputo A.A. (2002). Effect of splinting and interproximal contact tightness on load transfer by implant restorations. J. Prosthet. Dent..

[41] Hoshaw S.J., Brunski J.B., Cochran G.V. (1994). Mechanical loading of Brånemark implants affects interfacial bone modeling and remodeling. Int. J. Oral Maxillofac. Implants.

[42] Quirynen M., Naert I., Van Steenberghe D. (1992). Fixture design and overload influence marginal bone loss and future success in the Brånemark^®^ system. Clin. Oral Implants Res..

[43] de Medeiros R.A., Pellizzer E.P., Vechiato Filho A.J., Dos Santos D.M., da Silva E.V., Goiato M.C. (2016). Evaluation of marginal bone loss of dental implants with internal or external connections and its association with other variables: A systematic review. J. Prosthet. Dent..

[44] Vercruyssen M., Coucke W., Naert I., Jacobs R., Teughels W., Quirynen M. (2015). Depth and lateral deviations in guided implant surgery: An RCT comparing guided surgery with mental navigation or the use of a pilot-drill template. Clin. Oral Implants Res..

[45] Olate S., Lyrio M.C.N., de Moraes M., Mazzonetto R., Moreira R.W.F. (2010). Influence of diameter and length of implant on early dental implant failure. J. Oral Maxillofac. Surg..

